# Supplementary material for: Detecting anomalous electricity consumption with transformer and synthesized anomalies
Source: PeerJ Comput Sci. 2023 Dec 4;9:e1721. doi: 10.7717/peerj-cs.1721 (PMC10702936; doi:10.7717/peerj-cs.1721)
Supplement: Supplemental Information 1 [file peerj-cs-09-1721-s001.zip › code/Instructions_for_the_code.pdf]

Instructions for the code

data/ → directory for the experimental data.

figures/ → directory for storing figures.

transformer_detector.py → the Transformer model for detecting anomalous electricity consumption

cal_metrics.py → the script for calculating the evaluation metrics.

plt_ablation_results.py
plt_anomaly_types.py
plt_roc.py
plt_ts_input.py → the scripts for plotting figures in the paper.
